# A three-years assessment of *Ixodes ricinus*-borne pathogens in a French peri-urban forest

**DOI:** 10.1186/s13071-019-3799-7

**Published:** 2019-11-21

**Authors:** Emilie Lejal, Maud Marsot, Karine Chalvet-Monfray, Jean-François Cosson, Sara Moutailler, Muriel Vayssier-Taussat, Thomas Pollet

**Affiliations:** 1 UMR BIPAR, Animal Health Laboratory, INRA, ANSES, Ecole Nationale Vétérinaire d’Alfort, Université Paris-Est, Maisons-Alfort, France; 2 Laboratory for Animal Health, Epidemiology Unit, ANSES, University Paris Est, Maisons-Alfort, France; 3 UMR EPIA, VetAgro Sup, INRA, Université de Lyon, Université Clermont Auvergne, 63122 Saint-Genès-Champanelle, France; 4 Animal Health Department, INRA, Nouzilly, France

**Keywords:** Tick-borne pathogens, Dynamics, Temporal patterns, Pathogen co-occurrences

## Abstract

**Background:**

*Ixodes ricinus* is the predominant tick species in Europe and the primary pathogen vector for both humans and animals. These ticks are frequently involved in the transmission of *Borrelia burgdorferi* (*sensu lato*), the causative agents of Lyme borreliosis. While much more is known about *I. ricinus* tick-borne pathogen composition, information about temporal tick-borne pathogen patterns remain scarce. These data are crucial for predicting seasonal/annual patterns which could improve understanding and prevent tick-borne diseases.

**Methods:**

We examined tick-borne pathogen (TBP) dynamics in *I. ricinus* collected monthly in a peri-urban forest over three consecutive years. In total, 998 nymphs were screened for 31 pathogenic species using high-throughput microfluidic real-time PCR.

**Results:**

We detected DNA from *Anaplasma phagocytophilum* (5.3%), *Rickettsia helvetica* (4.5%), *Borrelia burgdorferi* (*s.l.*) (3.7%), *Borrelia miyamotoi* (1.2%), *Babesia venatorum* (1.5%) and *Rickettsia felis* (0.1%). Among all analysed ticks, 15.9% were infected by at least one of these microorganisms, and 1.3% were co-infected. Co-infections with *B. afzeli*/*B. garinii* and *B. garinii*/*B. spielmanii* were significantly over-represented. Moreover, significant variations in seasonal and/or inter-annual prevalence were observed for several pathogens (*R. helvetica*, *B. burgdorferi* (*s.l.*)*, B. miyamotoi* and *A. phagocytophilum*).

**Conclusions:**

Analysing TBP prevalence in monthly sampled tick over three years allowed us to assess seasonal and inter-annual fluctuations of the prevalence of TBPs known to circulate in the sampled area, but also to detect less common species. All these data emphasize that sporadic tick samplings are not sufficient to determine TBP prevalence and that regular monitoring is necessary.

## Background

Ticks are obligatory hematophagous arthropods and consequently, are one of the most important pathogen vectors [1–3]. Lyme borreliosis (LB) is the most commonly reported tick-borne disease (TBD) in the northern hemisphere and is caused by bacteria belonging to the *Borrelia burgdorferi* (*s.l.*) complex. In western Europe, *Ixodes ricinus* is known to be involved in the transmission of these bacteria to both humans and animals. This tick species has also been reported to be a vector for many other tick-borne pathogens (TBPs) with potentially significant consequences for human and animal health (*Anaplasma*, *Rickettsia*, *Bartonella*, *Babesia*, etc.) [4–9].

While multiple different pathogens have been identified and confirmed in *I. ricinus* ticks, very little is known about their seasonal and inter-annual variations. Time-series studies are thus crucial to understanding natural variability in microbial communities over time. Over the last decade, only a handful of surveys have assessed seasonal and monthly TBP variation patterns [10–14]. Although these results have heightened our general understanding of TBP dynamics, several of these studies were performed over short periods of less than two years, rendering it impossible to infer inter-annual discrepancies or to detect bias due to a particularly exceptional year. Only Coipan et al. [12] analysed several pathogenic genera in ticks sampled over more than two years. This study did demonstrate relationships between seasons and TBP prevalence (*Borrelia*, *Rickettsia*, *Anaplasma*, *Neoehrlichia* and *Babesia*) in questing tick populations. These variations were mainly attributed to the varying availability of reservoir hosts.

Tick density is also heavily influenced by the presence of suitable hosts, most notably wild ungulates that sustain adults, thus enabling tick population renewal [15, 16]. However, it is important to emphasise that immediate tick survival and questing activities are highly dependent on suitable and specific environmental conditions (temperatures between 8–24 °C and humidity of up to 80%). Simultaneously, several studies have investigated whether pathogen presence influences tick behaviour. Herrmann & Gern [17, 18] suggested that *I. ricinus* infected with *B. burgdorferi* (*s.l.*) can tolerate increased levels of desiccation, and Neelakanta et al. [19] demonstrated that *I. scapularis* infected with *Anaplasma phagocytophilum* are more resistant to cold. The presence of these TBPs could therefore enhance survival or questing activities of the infected ticks under challenging abiotic conditions, suggesting the existence of a potential link between pathogen prevalence in questing ticks and seasons.

Tick density and TBP prevalence can thus be influenced by several variables, and can therefore potentially fluctuate both seasonally and annually. Studying these dynamics is essential to better understanding and anticipating TBP risk.

Peri-urban forests containing both TBP-reservoir hosts and ticks, and which are highly frequented by people and their pets, represent a particularly interesting area to study tick and TBP dynamics. The Sénart forest, located to the south of Paris, harbours many large ungulates and abundant and diverse populations of other TBP reservoir hosts (bank voles, wood mice, Siberian chipmunks, roe deer, hedgehogs, etc.), and accommodates more than three million visitors every year. This forest is therefore particularly adapted to studying ticks and tick-borne pathogen dynamics.

In this study, we assessed the seasonal and inter-annual variability of *I. ricinus*-borne pathogens in the Sénart forest over three consecutive years (from April 2014 to May 2017), and determined whether any significant associations existed between these pathogens. We investigated a total of 31 pathogenic species (bacteria and parasites), belonging to 11 genera: *Borrelia*, *Anaplasma*, *Ehrlichia*, *Neoehrlichia* (only *N. mikurensis*), *Rickettsia*, *Bartonella*, *Francisella*, *Coxiella*, *Theileria*, *Babesia* and *Hepatozoon*.

## Methods

### Tick collection

**Fig. 1:**
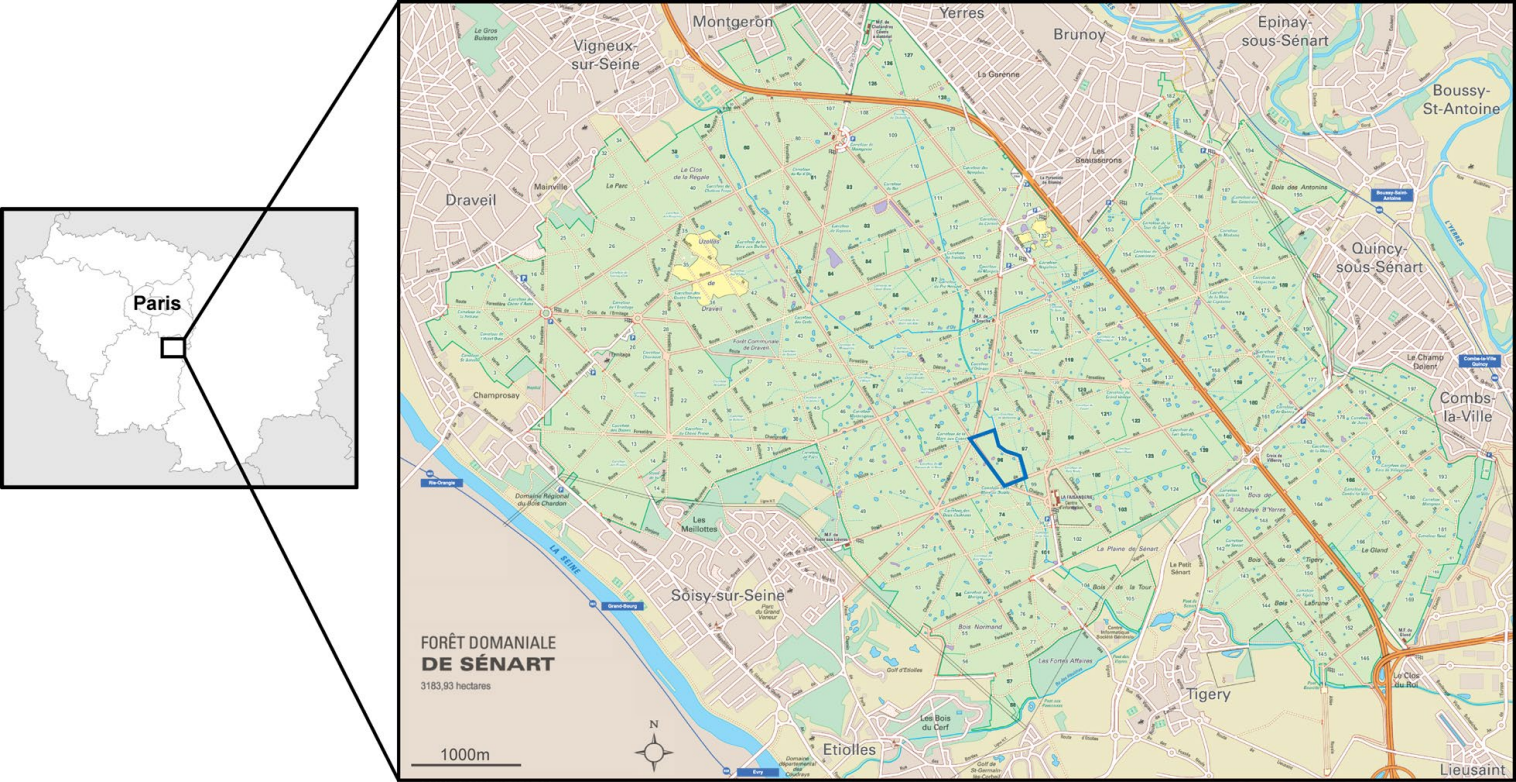
Sénart forest, location and parcel map. Sampling was made on the blue framed parcel

*Ixodes ricinus*, nymphs and adults, were monthly collected during three years, from April 2014 to May 2017, in the Sénart forest in the south of Paris. Ticks were collected between 10:00 h and 12:00 h. Samplings were performed by dragging [20] on 10 transects of 10 square meters, localized on the parcel 96 (48°39′34.6″N, 2°29′13.0″E, Fig. 1). Dragging was always performed 3 consecutive times on each transect by the same persons to limit sampling bias. The presence of *Dermacentor* spp. was occasionally reported but no investigation has been led further. The presence of *I.* *ricinus* larvae was also sometimes noticed. Due to their small size making individual DNA extraction and analysis difficult, we chose to not collect them. After morphological identification, ticks were stored at − 80 °C. In total 1167 ticks were collected.

### Tick washing, crushing and DNA extraction

Ticks were first washed once in ethanol 70% for 5 min and rinsed twice in sterile MilliQ water for 5 min. Ticks were then individually crushed in 375 µl of Dulbeccoʼs Modified Eagle Medium (DMEM) with decomplemented Foetal Calf Serum (10%) and six steel beads using the homogenizer Precellys^®^24 Dual (Bertin, Paris, France) at 5500× *rpm* for 20 s.

DNA extractions were then performed on 100 µl of tick crushing, using the DNA extraction kit NucleoSpin^®^ Tissue (Macherey-Nagel, Hoerdt, Germany), and following the standard protocol for human or animal tissue and cultured cells, from the step 2. DNA extracts were eluted in 50 µl of elution buffer and stored at − 20 °C until further use.

Two controls were performed: (i) the crushing control, corresponding to a DMEM tube in which crushing and DNA extraction were performed in the same conditions as on samples; and (ii) the extraction control, corresponding to the DNA extraction step without tick samples.

### Tick-borne pathogens detection

A high-throughput screening of the most common bacterial and parasite species known to circulate in ticks in Europe was performed. This allowed us to target simultaneously 31 pathogenic species, 7 genera and 1 phylum: the genus *Borrelia* and 8 *Borrelia* spp. (*B. burgdorferi* (*s.s.*), *B. afzelii*, *B. garinii*, *B. valaisiana*, *B. spielmanii*, *B. lusitaniae*, *B. bissettii* and *B. miyamotoi*); the genus *Rickettsia* and 6 *Rickettsia* spp. (*R. conorii*, *R. slovaca*, *R. massiliae*, *R. helvetica*, *R. aeshlimanii* and *R. felis*); the genus *Anaplasma* and 5 *Anaplasma* spp. (*A. phagocytophilum*, *A. platys*, *A. marginale*, *A. centrale* and *A. bovis*); the genus *Ehrlichia* and *E. canis*; *Neoehrlichia mikurensis*; the genus *Bartonella* and *B. henselae*; *Francisella tularensis*; *Coxiella burnettii*; the phylum Apicomplexa and 7 *Babesia* spp. (*B. divergens*, *B. microti*, *B. caballi*, *B. canis*, *B. venatorum*, *B. bovis* and *B. ovis)*, but also the two parasitic genera *Theileria* and *Hepatozoon*.

TBP DNA was detected using the BioMark™ real-time PCR system (Fluidigm, San Francisco, USA), a microfluidic system allowing to perform 48 or 96 real-time PCR reactions on 48 or 96 different samples as described in Michelet et al. [21] and Moutailler et al. [22]. Briefly, each sample and primers/probe set were included in individual wells. A pressure system allowed to load them on the chip *via* microchannels, in individual reaction chambers of 10 nl, where each sample will be individually mixed with each primer/probe set.

### Primers and probes

Primers and probes used for this analysis have been developed and validated by Michelet et al. [21] and Gondard et al. [23]. They have been designed to specifically amplify DNA from pathogens (bacteria and parasites) which are usually found in ticks in Europe. Their sequences, amplicon size, as well as targeted genes and pathogens are presented in Additional file 1: Table S1. Please note that, due to potential cross-reactions between primer/probe combinations (i.e. design) targeting *B. burgdorferi* (*s.s.*) and *B. spielmanii* with respectively *B. garinii*/*B. valaisiana* and *B. afzelii* DNA (described in [21]), positive samples for the two former species were considered as negative when associated to the latter. Therefore, potential associations between *B. burgdorferi* (*s.s.*)/*B. garinii*, *B. burgdorferi* (*s.s.*)/*B. valaisiana* and *B. spielmanii*/*B. afzelii* cannot be detected and the co-infection percentage may be under-estimated.

### DNA pre-amplification

DNA pre-amplifiations were performed using the TaqMan PreAmp Master Mix (Applied Biosystems, Illkirch, France). Basically, the different primer pairs, used for the real time PCR, were pooled combining equal volume of primers with a final concentration of 0.2 µM. For each sample, 1.25 µl of DNA extract was pre-amplified using the Perfecta PreAmp SuperMix reagent (1×) and the 0.2× pool (0.05 µM), in a final reaction volume of 5 µl. PCR cycle comprised a first cycle at 98 °C for 2 min, followed by 14 cycles with 2 steps, the first one at 95 °C for 10 s and the second one at 60 °C for 3 min. Pre-amplified DNA were then diluted (1:10) by addition of 45 µl of sterile deionised water before use.

### High throughput real time PCR

For each pre-amplified sample, the BioMark™ real-time PCR system (Fluidigm, San Francisco, USA) was used for high-throughput microfluidic real-time PCR amplification using the 48.48 microfluidic dynamic array (Fluidigm, San Francisco, USA). Amplifications were performed using FAM- and black hole quencher (BHQ1)-labeled TaqMan probes with TaqMan Gene Expression Master Mix in accordance with manufacturer’s instructions (Applied Biosystems, Illkirch, France). Thermal cycling conditions were used as follows: 95 °C for 5 min, 45 cycles at 95 °C for 10 s, 60 °C for 15 s, and 40 °C for 10 s. Data were acquired on the BioMark Real-Time PCR system and analysed using the Fluidigm Real-Time PCR Analysis software to obtain crossing point (CP) values. Three tick species controls (*I. ricinus*, *Dermacentor reticulatus*, *Dermacentor marginatus*), one negative water control and one positive *Escherichia coli* control were included in each chip.

### Nested PCR and sequencing

Samples that were positive either only for species design but not for the genus design or only for the genus design and not for the species design were all re-analysed by nested PCR. We used primer pairs allowing to target another gene that the one tested into the real-time PCR experiment. Their sequences, amplicon size, as well as targeted genes and pathogen genus are presented in Additional file 2: Table S2. Amplicons were sequenced by the Eurofins company and sequences analysed using the Bioedit software and compared to the database NCBI (National Center for Biotechnology Information) by sequence alignment using nucleotide BLAST (Basic Local Alignment Search Tool).

### Statistical analysis

#### TBP prevalences at the seasonal and multi-annual scale

Differences in TBP prevalences were tested within and between years by using a multivariable logistic regression model. We considered the calendar season level for the within-year variability. Seasons were considered as following: Winter (December to February); Spring (March to May); Summer (June to August); and Autumn (September to November). Logistic regression models were developed using the TBP status of each nymph as the outcome measure and season and year as explanatory variables. We performed four specific models for the following group/species of TBPs: (i) *B. burgdorferi* (*s.l.*) (considering *B. burgdorferi* (*s.s.*), *B. garinii*, *B. afzelii*, *B. valaisiana* and *B. spielmanii*); (ii) *B. miyamotoi*; (iii) *A. phagocytophilum*; and (iv) *R. helvetica*. The models were constructed from a generalized linear model option (GLM) [24] using a binomial distribution (logit link). Model assessment was based on Akaikeʼs information criterion (AIC). Results were expressed as odds ratios (OR) and 95% confidence intervals. Statistical computations were performed in R 3.5.1. [25].

#### Statistical modelling of tick-borne pathogen associations

We tested the associations between the TBP species that belonged to the co-infection profiles of nymphs found in this study. We used the association screening approach [26]. For a given number of pathogen species tested (NP), the number of possible combination (NC) was calculated as NC = 2^NP^. Assuming similar pathogen prevalence as those observed, a simulated dataset was built as a presence/absence matrix with hosts in lines and pathogen combinations in columns. With 5000 simulations, we obtained the NC statistic distributions. We estimated a 95% confidence interval to obtain a profile that includes simultaneously all the combinations. From this profile, we inferred for each combination two quantiles, *Qinf* and *Qsup*. A global test was based on the 95% confidence envelope. When H0 was rejected, the local tests were based on the NC confidence intervals (*Qinf*–*Qsup*) [26].

## Results

### Tick temporal dynamics

**Fig. 2:**
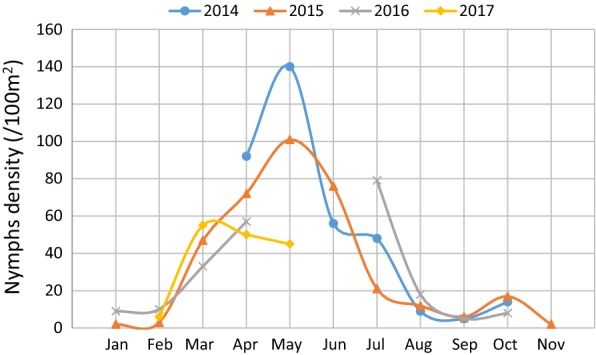
*Ixodes ricinus* nymphs monthly density (per100 m^2^) in 2014, 2015, 2016 and 2017. Ticks were sampled from April 2014 to May 2017. Please note that May and June 2016 were unfortunately not sampled

From April 2014 to May 2017, a total of 1167 *I. ricinus* ticks were collected in the Sénart forest in the south of Paris (Fig. 1). Please note that May and June 2016 were unfortunately not sampled due to logistic issues. Collected ticks were composed of 1098 nymphs, 35 females and 34 males. Adults were sporadically detected all over the three years; due to their low total abundance (more than 10-fold less compared with nymphs), we decided to focus our temporal analysis on nymphs. The temporal dynamics of nymph densities over the three years is shown in Fig. 2. Nymph densities followed similar patterns from one year to another, with a main peak of activity observed every year in spring, a strong decrease during summer and a second peak, smaller, observed in Autumn (Fig. 2). In January and February, the average densities were less than 10 questing nymphs/100 m^2^. A clear rise was observed from March to May reaching an average peak of 95 nymphs/100 m^2^ in May. We then observed a decrease in summer up to a minimum average of 5 nymphs/100 m^2^ in September. The nymph densities increased slightly in October to reach an average of 13 nymphs/100 m^2^, before finally decreasing in November (2 nymphs/100 m^2^, sampled in 2015).

### Detected pathogens and their prevalence in tick population

Due to technical problems, DNA was extracted and analysed only from 1044 nymphs among the 1098 previously mentioned. A total of 46 were negative for at least one positive control and thus have been removed from the analysis. From the 998 remaining DNA samples, 15.9% (95% CI: 13.7–18.3%) were positive for at least one tested pathogen, which belong to three bacterial and one protozoan genera: *Anaplasma*, *Borrelia*, *Rickettsia* and *Babesia* (Table 1).

**Table 1:** Summary table of the TBP detection study results

Y	M	NN	*B. bu* (*s.s*.)	*B. ga*	*B. af*	*B. va*	*B. spi*	*B. bu* (*s.l.*)	*B. mi*	*Bo.* spp.	*A. ph*	*A.* spp.	*R. he*	*R. fe*	*R.* spp.	*B. ve*	*Ba. spp*	2	3	TM
2014	Apr	89	0	0	0	0	0	0	0	0	10	10	7	0	7	0	1	0	0	18
	May	127	1	0	0	0	0	1	1	2	12	12	2	0	2	4	4	2	0	18
	Jun	54	0	1	1	1	0	1	0	1	4	4	9	1	10	2	2	1	1	16
	Jul	38	0	1	0	0	1	1	0	1	6	6	1	0	1	0	0	1	0	8
	Aug	9	0	0	0	0	0	0	0	0	0	0	1	0	1	0	0	0	0	1
	Sep	5	1	0	0	0	0	1	0	1	0	0	0	0	0	1	1	0	0	2
	Oct	13	0	0	0	1	0	1	0	1	1	1	0	0	0	0	0	0	0	2
2015	Jan	2	0	0	0	0	0	0	0	0	0	0	0	0	0	0	0	0	0	0
	Feb	3	0	0	1	0	0	1	0	1	0	0	0	0	0	0	0	0	0	1
	Mar	43	0	3	1	1	1	3	3	6	1	1	5	0	5	0	0	1	1	12
	Apr	69	2	0	0	0	0	2	0	2	3	4	4	0	4	0	0	0	0	10
	May	88	0	0	0	0	0	0	1	1	1	1	3	0	3	2	2	0	0	7
	Jun	78	1	0	0	0	1	2	2	4	2	2	1	0	1	2	2	1	0	8
	Jul	21	0	2	2	0	0	3	1	4	0	0	0	0	0	0	0	1	0	4
	Aug	6	0	0	0	0	0	0	0	0	0	0	0	0	0	0	0	0	0	0
	Sep	6	0	0	0	0	0	0	0	0	0	0	0	0	0	0	0	0	0	0
	Oct	17	4	0	0	0	0	4	0	4	0	0	0	0	0	0	0	0	0	4
	Nov	2	0	0	0	0	0	0	1	1	0	0	0	0	0	0	0	0	0	1
2016	Jan	9	0	0	1	0	0	1	0	1	0	0	1	0	1	0	0	0	0	2
	Feb	10	0	0	0	0	0	0	0	0	0	0	1	0	1	0	0	0	0	1
	Mar	26	2	1	2	0	0	4	0	4	1	1	0	0	0	0	0	1	0	5
	Apr	33	0	0	0	1	0	1	0	1	0	0	2	0	2	0	0	0	0	3
	Jul	78	2	2	2	0	1	5	0	5	2	2	4	0	4	1	1	1	1	11
	Aug	11	2	0	0	0	0	2	0	2	0	0	2	0	2	0	0	1	0	3
	Sep	5	0	0	0	0	0	0	0	0	0	0	1	0	1	0	0	0	0	1
	Oct	6	0	0	0	0	0	0	1	1	0	0	0	0	0	0	0	0	0	1
2017	Feb	6	0	0	0	0	0	0	1	1	0	0	0	0	0	0	0	0	0	1
	Mar	53	0	1	0	1	0	2	0	2	3	3	0	0	0	2	2	0	0	7
	Apr	50	0	0	1	1	0	2	0	2	3	3	0	0	0	1	1	0	0	6
	May	41	0	0	0	0	0	0	1	1	4	4	1	0	1	0	0	0	0	6
	T	998	15	11	11	6	4	37	12	49	53	54	45	1	46	15	16	10	3	159
	NM	30	8	7	8	6	4	18	9	23	14	14	16	1	16	8	9	9	3	27

*Abbreviations*: Y, year; M, month; NN, number of analysed nymphs; TM, total number of positive samples per month; *B. bu* (*s.s.*), *B. burgdorferi* (*s.s.*); *B. ga*, *B. garinii*; *B. af*. *B. afzelii*; *B. va*, *B. valaisiana*; *B. spi*, *B. spielmanii*; *B. bu* (*s.l.*), *B. burgdorferi* (*s.l.*); *B. mi*, *B. miyamotoi*; *Bo.* spp., *Borrelia* spp; *A. ph*, *A. phagocytophilum*; *A.* spp., *Anaplasma* spp.; *R. he*, *R. helvetica*; *R. fe*, *Rickettsia felis*; *R.* spp., *Rickettsia* spp; *B. ve*, *B. venatorum*; *Ba.* spp., *Babesia* spp.; 2, co-infections with 2 pathogens; 3, co-infections with 3 pathogens; T, total number of positive samples; NM, number of positive months

Pathogens belonging to the genus *Anaplasma* were detected in 5.4% (95% CI: 4.1–7.0%) of collected ticks. Most of them were positive for *A. phagocytophilum* (5.3% of all the samples) and one DNA sample was only positive for the primers/probe combination specific to *Anaplasma* spp. This sample was confirmed by nested PCR and the amplicon was then sequenced. The BLAST analysis on NCBI showed that this sequence matched at 99% of identity with four different *Anaplasma* species (*A. phagocytophilum*, *A. marginale*, *A. ovis* and *A. centrale*). Therefore, this sample was only considered as positive for *Anaplasma* spp.

Two species of *Rickettsia* were detected in questing *I. ricinus* nymphs. *Rickettsia helvetica* was the most prevalent and was detected in 4.5% (95% CI: 3.3–6.0%) of nymphs. *Rickettsia felis* was detected in only one nymph (0.1%, 95% CI: 0.003–0.6%). The presence of *R. felis* DNA was confirmed by nested PCR and sequencing of the *ompB* gene. The obtained sequence (GenBank: MN267050) matched with the corresponding gene sequence of *R. felis* (GenBank: GU182892.1) with an identity of 100% and query cover of 100%.

The genus *Borrelia* was represented by six different species detected in 4.9% (95% CI: 3.7–6.4%) of the surveyed nymphs. Five belonged to the LB group (3.7%, 95% CI: 3.7–6.4%), including *B. burgdorferi* (*s.s.*) (1.5%, 95% CI: 0.8–2.5%), *B. garinii* (1.1%, 95% CI: 0.6–2.0%), *B. afzelii* (1.1%, 95% CI: 0.6–2.0%), *B. valaisiana* (0.6%, 95% CI: 0.2–1.3%) and *B. spielmanii* (0.4%, 95% CI: 0.1–1.0%). DNA of *Borrelia miyamotoi*, belonging to the relapsing fever group, was detected in 1.2% (95% CI: 0.6–2.1%) of the collected nymphs.

DNA from two species of *Babesia* were detected in questing nymph with the microfluidic PCR: *Babesia venatorum* (1.5%, 95% CI: 0.8–2.5%) of ticks and *Babesia divergens* (0.1%, 95% CI: 0.003–0.6%), detected in one tick. A deeper investigation of the *B. divergens*-positive sample, by nested PCR on the *18S* rRNA gene and amplicon sequencing (GenBank: MN296295), allowed us to finally identify the DNA of *B. capreoli*, a species closely related to *B. divergens*, circulating in wild ruminants and unable to infect human and cattle erythrocytes [27].

### Temporal patterns of TBPs in *I. ricinus* nymphs

#### TBP prevalence at the monthly scale

In the following paragraph and corresponding figures, prevalences are those obtained for months with at least nine collected ticks.

**Fig. 3:**
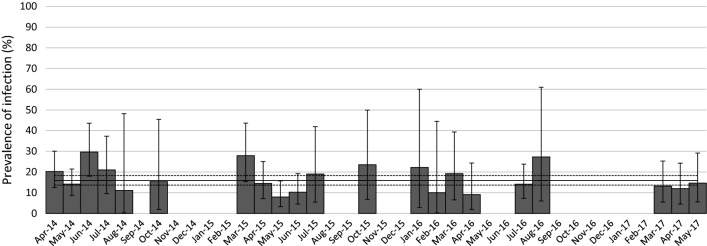
Nymph infection rate per month for at least one tested pathogen. Months with less than nine nymphs sampled have not been considered for percentage calculation. Error bars represent confidence intervals

**Fig. 4:**
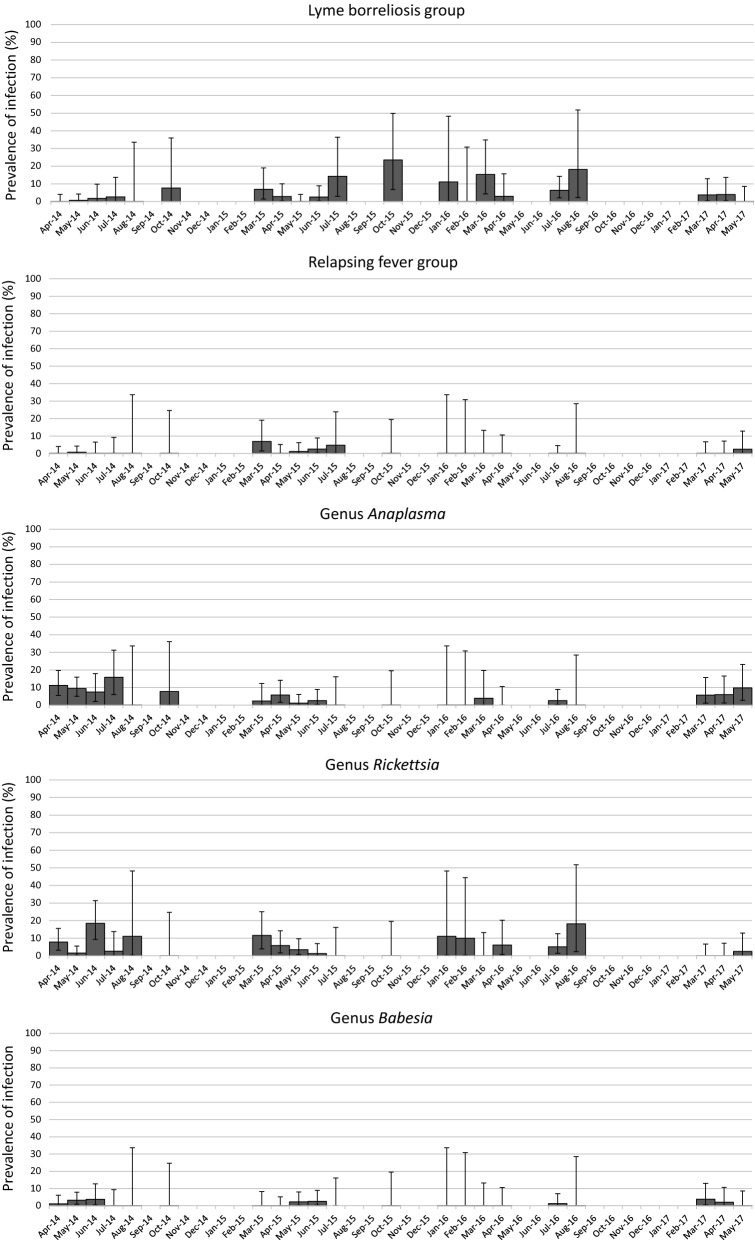
Nymph infection rate and confidence intervals per month for the different TBPs. Months with less than nine nymphs sampled have not been considered. Error bars represent confidence intervals

Global infection rates fluctuated from 8% (95% CI: 3.3–15.7%) in May 2015 to 29.6% (95% CI: 18.0–43.6%) in June 2014 (Fig. 3). At the genus level, variations in TBP prevalences and the number of months for which at least one tick was positive for each tested TBPs are presented in Fig. 4 and Table 1.

DNA from pathogens belonging to both genera *Rickettsia* and *Anaplasma* were detected in 16 and 14 of the 30 sampled months, respectively. When detected, prevalences fluctuated from 1.3% (95% CI: 0.03–6.9%) (June 2015) to 18.5% (95% CI: 9.3–31.4%) (June 2014) for *Rickettsia* and from 1.1% (95% CI: 0.03–6.2%) (May 2015) to 15.8% (95% CI: 6.0–31.3%) (July 2014) for *Anaplasma*. Both genera are mainly represented by one species: *R. helvetica* and *A. phagocytophilum* that are the most frequently detected species (16 and 14 of the 30 sampled months, respectively). These two species were found each year.

DNA from members of the genus *Borrelia* was detected in 23 of the 30 sampled months. This bacterial genus displayed the highest variability with prevalences fluctuating from 1.1% (95% CI: 0.03–6.2%) (May 2015) to 23.5% (95% CI: 6.8–49.9%) (October 2015). DNA from members of the LB group was detected in 18 of the 30 sampled months with prevalences ranging from 0.8% (95% CI: 0.03–6.2%) in May 2014 to 23.5% (95% CI: 6.8–49.9%) in October 2015. The most frequently identified species were *B. burgdorferi* (*s.s.*) (8/30 sampled months), *B. afzelii* (8/30) and *B. garinii* (7/30). DNA from these species was regularly detected over the three studied years. Conversely, *B. valaisiana* (6/30) and *B. spielmanii* (4/30) DNA was not detected during 11 (from April 2015 to March 2016) and 9 (from July 2015 to April 2016) consecutive months, respectively. *Borrelia miyamotoi* (relapsing fever group) DNA was detected 9 times over the 30 sampled months with prevalences ranging from 0.8% (95% CI: 0.02–4.3%) in May 2014 to 7% (95% CI: 1.5–19.1%) in March 2015.

For parasites, DNA from the genus *Babesia* was detected in 9 months out of 30 sampled months. Prevalences presented the lowest variability ranging from 1.1% (95% CI: 0.03–6.1%) in April 2014 to 3.8% (95% CI: 0.5–13.0%) in March 2017 (Fig. 4). The main detected species was *B. venatorum* that was detected 9 times over 30 samplings and not detected during 9 consecutive sampled months, from June 2015 to April 2016.

#### TBP prevalences at the seasonal and multi-annual scale

In order to determine if the prevalence of TBPs was different within and between years, a multivariable logistic regression model was performed. The spring season and the year 2014 have been considered as references for the seasonal and yearly effect, respectively. Because some TBPs had too low prevalences in the nymph population (producing unreliable statistics), analyses were only performed on the most prevalent TBPs: *A. phagocytophilum*, *R. helvetica*, *B. burgdorferi* (*s.l.*), *B. miyamotoi* and *B. venatorum*.

**Fig. 5:**
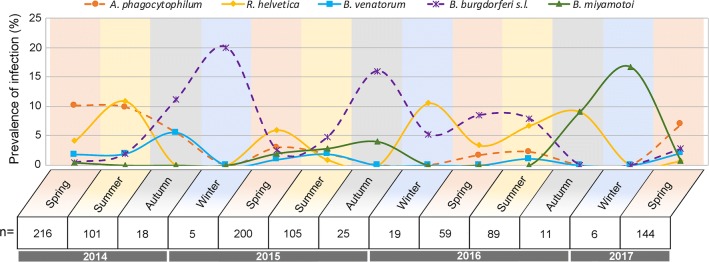
Percentage of positive nymphs per season for the most prevalent TBPs. Winter (January to February, pastel blue background); Spring (March to May, pastel orange background); Summer (June to August, pastel yellow background); Autumn (September to November, light grey background). Abbreviation: n, number of analysed ticks

Significant differences, in terms of prevalence, were observed at the seasonal scale (Table 2, Fig. 5) for *R. helvetica* (higher in summer compared to spring), *B. burgdorferi* (*s.l.*) (higher in autumn compared to spring) and *B. miyamotoi* (higher in winter than in spring). Please note that the smallest number of sampled ticks (30 in total) was found in winter, and that the difference observed for *B. miyamotoi* in winter corresponded to only one infected tick collected in February 2017.

**Table 2:** Multivariable logistic regression models assessing the seasonal and yearly TBP prevalence variations in nymphs. Odds ratios and their associated 95% confidence intervals obtained from the best model of TBP seasonal and yearly prevalence in questing nymphs

Model	TBP	Variable	Odds ratio	95% CI		Significant difference
				Low	High	
1	*B. burgdorferi* (*s.l.*)	Spring	Ref			
		Autumn	4.53	1.50	12.49	Yes
		Summer	1.69	0.75	3.89	No
		Winter	1.73	0.25	7.01	No
		2014	Ref			
		2015	2.93	1.12	9.14	Yes
		2016	4.48	1.60	14.53	Yes
		2017	2.45	0.57	9.95	No
2	*B. miyamotoi*	Spring	Ref			
		Autumn	0	na	8.3275E+218	No
		Summer	0	na	2.26397E+88	No
		Winter	28.60	1.03	800.00	Yes
3	*A. phagocytophilum*	2014	Ref			
		2015	0.20	0.08	0.42	Yes
		2016	0.16	0.04	0.45	Yes
		2017	0.65	0.30	1.32	No
4	*R. helvetica*	Spring	Ref			
		Autumn	0	0	6.7759E+11	No
		Summer	3.10	1.27	7.85	Yes
		Winter	0	na	1.1447E+145	No
		2014	Ref			
		2015	1.34	0.54	3.39	No
		2016	0.81	0.12	3.24	No
		2017	0.16	0.01	0.87	Yes

*Abbreviations*: CI, confidence interval; ref, reference; na, not applicable

Significant differences were also observed between years for bacteria belonging to the complex *B. burgdorferi* (*s.l.*) with higher prevalence in 2015 and 2016 compared to 2014; for *A. phagocytophilum*, with lower prevalence in 2015 and in 2016 compared to 2014 and for *R. helvetica*, with lower prevalence in 2017 than in 2014. However, please note that samplings were only performed from January to May in 2017. No significant differences were observed in relation to season or year for *B. venatorum*.

### Pathogen associations

Among all the sampled ticks, 1% (95% CI: 0.5–1.8%) were co-infected with two pathogens and 0.3% (95% CI: 0.006–0.8%) were co-infected with three pathogens. Eight different co-infection profiles were found (Table 3). In most of cases (7/13), these co-infections concerned species belonging to the genus *Borrelia*: *B. garinii* + *B. afzelii*; *B. garinii* + *B. spielmanii*; *B. garinii* + *B. afzelii* + *B. valaisiana* and *B. garinii* + *B. valaisiana* + *B. spielmanii*. Co-infection profiles with species belonging to different genera were also observed: *A. phagocytophilum* + *B. venatorum*; *A. phagocytophilum* + *R. helvetica*; *B. burgdorferi* (*s.s.*) + *R. helvetica* and *B. garinii* + *B. afzelii* + *R. helvetica*. All these associations between pathogens were tested using the association screening approach of Vaumourin et al. [26]. Compared to a random analysis, no associations were found to be underrepresented while two were overrepresented: the first one between *B. garinii* and *B. afzelii* (observation = 3; minimum expected = 0; maximum expected = 2), and the second one between *B. garinii* and *B. spielmanii* (observation = 2; minimum expected = 0; maximum expected = 1).

**Table 3:** Summary table of the reported co-infection profiles

*B. bu* (*s.s.*)	*B. ga*	*B. af*	*B. va*	*B. spi*	*A. ph*	*R. he*	*B. ve*	Co-occurrence number
	×	×						3
	×			×				2
	×	×	×					1
	×		×	×				1
					×		×	3
					×	×		1
×						×		1
	×	×				×		1

*Abbreviations*: *B. bu* (*s.s.*); *B. burgdorferi* (*s.s.*); *B. ga*, *B. garinii*; *B. af*, *B. afzelii*; *B. va*, *B. valaisiana*; *B. spi*, *B. spielmanii*; *A. ph*, *A. phagocytophilum*; *R. he*, *R. helvetica*; *B. ve*, *B. venatorum*

## Discussion

### *Ixodes ricinus* density and seasonal dynamics

This three-year survey demonstrated a clear seasonal pattern in *I. ricinus* density, with a marked peak of questing nymphs in spring and a smaller peak in autumn. Low, but present activity was detected in winter, as has been observed in Germany [28]. In addition to these general patterns, some unexpected data were observed, the most striking being no peak activity in spring 2017 (April and May) with tick densities very similar to those recorded in March. Abiotic factors such as temperature, relative humidity and rainfall, or fluctuating host numbers in the sampling area are known to influence questing tick abundance and activity patterns [29–34] and could explain these unusual observations. It is important to note that 2017 was distinguished by an abnormally wet March, with total rainfall much higher than that recorded in previous years in the same area (71.3, compared to 11.2, 33.6 and 61.7 mm rain/month in 2014, 2015 and 2016, respectively). Interestingly, the increased March rainfall was followed by an April drought (7.9 mm of rain/month in 2017, compared to 48.4, 27.2 and 66.2 mm rain/month in 2014, 2015 and 2016, respectively) (rainfall data estimated from the Orly station, Metéo-France data; https://donneespubliques.meteofrance.fr/?fond=produit&id_produit=90&id_rubrique=32). These unusual meteorological characteristics could explain the stable tick density from March to May 2017. Thereby, this finding clearly shows that the bimodal tick activity pattern usually observed during this study can exceptionally change with unusual environmental conditions, reinforcing the importance of regular monitoring.

### *Ixodes ricinus*-borne pathogen composition and prevalence over the three years

Most of the detected pathogen species corresponded to microorganisms known to circulate in the Western Palaearctic [35–42]. However, several species belonging to the genera *Bartonella* and *Francisella* previously reported in the studied area [40, 43], were not detected. The most prevalent pathogen species were *A. phagocytophilum* (5.4% of the examined nymphs), *R. helvetica* (4.5%), and *B. burgdorferi* (*s.l.*) (3.7%). Both high- and low-prevalence TBPs were consistently detected in the sampling area for the duration of the study. Although prevalences varied between different TBPs, and some were not detected for long periods, they were all detected recurrently. Continued detection is consistent with the year-round presence of reservoir hosts in the sampling area (wood mice, bank voles, Siberian chipmunks, roe deer, common blackbird, European robin, song thrush, etc.) [34, 44, 45]. The continued presence of reservoir hosts could facilitate the circulation of dominant species, and maintain, even at low rates, less prevalent pathogen species that may not be detected by a single sampling. This does support to regularly studying TBP temporal dynamics, to improve the assessment of their prevalence.

We also detected in a single tick, the DNA of the emergent human pathogen *R. felis*. Its detection is particularly unexpected as this bacterium is known to be mainly transmitted from cat to cat *via* fleas, with human contamination arising from cat or flea bites. As we only detected DNA from *R. felis*, we cannot exclude that this detection could correspond to remnant DNA from the previous blood meal. Nevertheless, several studies have already detected the presence of *R. felis* or *R. felis*-like organisms in hematophagous arthropods (reviewed in [46, 47]), including in ticks collected from natural environments [48], and notably in two studies performed on questing *I. ricinus*, including one based on RNA detection [49, 50]. Rarely investigated in studies dealing with TBPs, the repeated detection of *R. felis* should encourage increased surveillance for this spotted fever-causing pathogen in humans. Finally, all these findings suggest that a unique sampling would certainly not facilitate the detection of this pathogen, again highlighting the importance of collecting and analysing ticks at a large temporal scale.

*Babesia divergens* and *B. capreoli* are two closely related species. During this study, we found out that the design initially used to detect *B. divergens* actually also enabled detection of DNA of *B. capreoli*. While *B. divergens* is responsible of babesiosis in humans and cattle, *B. capreoli* is only able to colonize erythrocytes from deer, therefore presenting no threat for humans or livestock [27]. These results emphasise the importance of confirmation and careful interpretations of microfluidic real time PCR [51].

### Seasonal and inter-annual dynamics of *I. ricinus*-borne pathogens

Improving the prevention of TBDs requires a better understanding of their temporal, and in particular, their seasonal dynamics. However, only a few studies have addressed these issues during a minimum three-year period [12, 13]. As ticks were collected monthly for over three years in this study, we detected significant seasonal or annual infection rate fluctuations for four TBPs: *R. helvetica*, *B. burgdorferi* (*s.l.*), *B. miyamotoi* and *A. phagocytophilum*. Note that the statistically significant highest prevalence of *B. miyamotoi* in winter is only due to the detection of one positive tick sampled during winter in 2017. In our opinion, this result alone is insufficient to presume that *B. miyamotoi* have an increased winter prevalence. However, we can observe that even if very few ticks are questing during these periods, they may carry TBPs.

While significant seasonal and annual differences were observed for *B. miyamotoi* and *A. phagocytophilum*, respectively, the presence of *R. helvetica* and *B. burgdorferi* (*s.l.*) varied significantly according to both seasons and years. None of these microorganisms followed a similar pattern. Comparing our results to the multi-annual studies previously mentioned, we observed that only *R. helvetica* presented similar seasonal patterns [12]. This finding again emphasises how the season, the year or the sampling area can influence TBP presence and prevalence in questing tick populations.

The most common explanation for temporal variations in TBP prevalences is the variable availability of reservoir hosts during tick previous feeding stage. This hypothesis was already suggested by Coipan et al. [12] who observed that several micro-organisms, assumed to share the same reservoir host, also presented similar seasonal patterns. Because the tick life-cycle is fundamentally linked to its host, any changes to the available host spectrum will undoubtedly influence TBP prevalence in the tick community [52]. However, because the entire tick life-cycle is multi-annual, it is difficult anyway to know if nymphs questing at the same time did perform their previous blood meal at the same period. The same generation of questing nymphs could come from larvae that would have fed at a different moment and thus potentially upon different host species. An alternative hypothesis, based on both the presence of pathogens and tick physiology, could also explain these patterns. Carrying certain TBPs was shown to improve tick resistance to challenging abiotic conditions. Herrmann & Gern [17, 18] demonstrated that ticks carrying *Borrelia* species exhibited higher survival rates in desiccating conditions and a lower tendency to move to favourable conditions for maintaining water balance than non-infected ticks. This was associated to a higher reserve of energy in *Borrelia-*infected ticks [53] which would therefore exhibit higher resistance capacities to hydric stress notably. This hypothesis would explain the higher prevalence of *Borrelia*-infected questing ticks observed during or after the summer period in the present study and in those of Coipan & Takken [12, 13]. Similarly, Neelakanta et al. [19] demonstrated a higher expression of the *iafgp* gene, coding for an antifreeze glycoprotein, in *A. phagocytophilum*-infected ticks. This thus conferred to ticks a stronger resistance to cold that could lead to higher prevalence of *A. phagocytophilum*-infected questing ticks during or just after the winter. This hypothesis was not consistent with our data as *A. phagocytophilum* was not observed in greater prevalence during the cold seasons of our study.

Our results, in combination with those from the literature, support the hypothesis that TBP prevalence is influenced by both biotic and abiotic factors, and reiterate that sporadic samplings are insufficient to assess it.

### Pathogen co-occurrence

Tick co-infections are being identified more and more frequently [22, 42, 50, 54–58]. Clinical co-infections with several TBPs are commonly reported [59–61] and are known to affect both disease symptoms and severity [62, 63]. It is thus essential to investigate TBP associations in ticks, to better identify potential clinical co-infections and to improve epidemiological knowledge of TBDs.

In this longitudinal three-year study, two TBP associations were significantly overrepresented compared to a random distribution: the first one was between *B. garinii* and *B. afzelii*, as has been previously observed in studies using similar detection tools [22, 42], or different methods (*16S* rRNA gene sequencing [64]); the second one was between *B. garinii* and *B. spielmanii*. Interestingly, these findings contrast with published results on *I. ricinus* TBPs. While performing a meta-analysis on data published from 2010 to 2016, Strnad et al. [65] observed a negative correlation between *B. garinii* and *B. afzelii*. Similarly, Herrmann et al. [66] also detected a negative co-occurrence between these two species following an analysis of 7400 nymphs collected over three years. These results are coherent considering the host specificity of these *Borrelia* species. Indeed, *B. garinii* does not share the same reservoir host (birds) than *B. afzelii* or *B. spielmanii* (wood mice and bank voles, or hazel and garden dormice) [67–72], and none of these species are known to be transmitted transovarially.

Although the associations we identified were statistically “overrepresented”, in fact we only observed one additional association than the fixed overrepresentation threshold (i.e. observed associations = 3 and 2; minimum expected = 0 and 0; maximum expected = 2 and 1; for *B. garinii* + *B. afzelii* and *B. garinii* + *B. spielmanii* associations, respectively). This indicates that caution should be applied when drawing conclusions about permanent associations between these different bacteria in ticks. Several different hypotheses could potentially explain these associations in the same nymph. First, hosts are likely to carry several adjacent feeding ticks. This phenomenon, known as co-feeding, could promote pathogen exchange between ticks even in the absence of systemic host infection [73]. Secondly, as discussed by van Duijvendijk et al. [74], when bloodmeals are disrupted due to host grooming, immune response or death, ticks may feed on more than one host to completely engorge, and consequently be exposed to several pathogens. Thirdly, despite these TBP species segregating between bird and rodent hosts, all of them have been detected in hedgehogs [75, 76], and *B. afzelii* and *B. garinii* have been simultaneously detected in one Siberian chipmunk [45]. Both of these mammals were found to host a large number of tick larvae [45, 77], and Siberian chipmunks have been reported to induce higher *B. burgdorferi* (*s.l.*) infection rates in nymphs, compared to bank voles and wood mice [45] in the Sénart forest. A last hypothesis might be that our analytical methods do not enable distinguishing the rodent-circulating *B. garinii* OspA serotype 4 (corresponding to *B. bavariensis*) [78] from other *B. garinii* serotypes.

Associations between *B. garinii* and *B. valaisiana* are frequently reported, which is not surprising as these species share the same reservoir host [79]. This association was the most common TBP association in a meta-analysis of literature published between 1984 and 2003 [80], and has been reported several times recently [11, 66, 81]. While we observed this association twice, both times in association with a third *Borrelia* species, either *B. afzelii* or *B. spielmanii*, it was not significantly overrepresented compared to a random distribution. Among the three previously mentioned studies, only Herrmann et al. [66] demonstrated that this association was overrepresented when compared to a randomly sampled analysis. However, our study was performed on a much smaller dataset (998 *vs* 7400 analysed nymphs), with a halved co-infection percentage (1.3 *vs* 3%), indicating that our statistical analysis may be less powerful, which could explain why this association was not detected.

These contrasting tick pathogen association results highlight the complexity in clearly identifying pathogen associations in field-collected ticks. Several other parameters can also potentially influence pathogen association (host spectrum within the studied area, sample size influencing analytical statistical power, identification bias, etc.). In this context, performing investigations under controlled conditions (suitable TBP growing and tick breeding systems, etc.) will be a crucial future step to experimentally test these different associations and improve our knowledge of TBP co-occurrences.

## Conclusions

This three-year study of *I. ricinus-*borne pathogens (i) identified several TBPs previously reported in the area, consistent with reservoir host availability; (ii) allowed the surprising detection of *R. felis* DNA, a microorganism rarely reported in questing ticks; (iii) highlighted significant variations in seasonal and inter-annual pathogen prevalence; and (iv) identified several unexpected co-occurrences between pathogens belonging to the *B. burgdorferi* (*s.l.*) complex. All these results represent another step towards understanding the TBP ecology and emphasise the need to perform longitudinal studies, particularly because the main factors that are supposed to influence tick and TBP ecology could change in the next few years with climate changes. Associated to other factors such as host information or meteorological measures, this kind of data is crucial to allow a better understanding of TBP ecology and TBD epidemiology.

## Supplementary information

**Additional file 1: Table S1.** Targeted genes, amplicon size, primers and probe sequences used for TBP and tick species detection.

**Additional file 2: Table S2.** Targeted genes and primer sequences used for results confirmation.

## Data Availability

Data supporting the conclusions of this article are provided within the article. Sequences for *Babesia capreoli* and *Rickettsia felis* were submitted to the GenBank database under the accession numbers MN296295 and MN267050, respectively. The datasets used and/or analysed during the present study are available from the corresponding author upon reasonable request.

## Abbreviations

TBP: tick-borne pathogen
TBD: tick-borne disease
LB: Lyme borreliosis
*s.l.*: *sensu lato*
*s.s.*: *sensu stricto*
DMEM: Dulbeccoʼs Modified Eagleʼs Medium
NCBI: National Center for Biotechnology Information
BLAST: Basic Local Alignment Search Tool
GLM: generalized linear model
AIC: Akaikeʼs Information Criterion
OR: odds ratio
NP: number of pathogen species tested
NC: number of possible combinations
iafgp: *Ixodes scapularis* antifreeze glycoprotein
