# Supplementary material for: A three-years assessment of Ixodes ricinus-borne pathogens in a French peri-urban forest
Source: Parasit Vectors. 2019 Nov 21;12:551. doi: 10.1186/s13071-019-3799-7 (PMC6873405; doi:10.1186/s13071-019-3799-7)
Supplement: Supplementary file 1 — Additional file 1: Table S1. Targeted genes, amplicon size, primers and probe sequences used for TBP and tick species detection. [file 13071_2019_3799_MOESM1_ESM.pdf]

Additional file 1: Table S1. Targeted genes, amplicon size, primers and probe sequences used for TBP and tick species detection.

Targeted TBP	Targeted gene	Identity	Sequence	Amplicon size (bp)	Reference
Borrelia burgdorferi	rpoB	Forward<br>Reverse<br>Probe	GCTTACTCACAAAAGGCGTCTT<br>GCACATCTCTTACTTCAAATCCT<br>AATGCTCTTGGACCAGGAGGACTTTCA	83	[21]
Borrelia garinii	rpoB	Forward<br>Reverse<br>Probe	TGGCCGAACTTACCCACAAAA<br>ACATCTCTTACTTCAAATCCTGC<br>TCTATCTCTTGAAAGTCCCCCTGGTCC	88	[21]
Borrelia afzelii	fla	Forward<br>Reverse<br>Probe	GGAGCAAATCAAGATGAAGCAAT<br>TGAGCACCCTCTTGAACAGG<br>TGCAGCCTGAGCAGCTTGAGCTCC	116	[21]
Borrelia valaisiana	ospA	Forward<br>Reverse<br>Probe	ACTCACAAATGACAGATGCTGAA<br>GCTTGCTTAAAGTAACAGTACCT<br>TCCGCCTACAAGATTTCCTGGAAGCTT	135	[21]
Borrelia lusitaniae	rpoB	Forward<br>Reverse<br>Probe	CGAACTTACTCATAAAAGGCGTC<br>TGGACGTCTCTTACTTCAAATCC<br>TTAATGCTCTCGGGCCTGGGGGACT	87	[21]
Borrelia spielmanii	fla	Forward<br>Reverse<br>Probe	ATCTATTTTCTGGTGAGGGAGC<br>TCCTTCTTGTTGAGCACCTTC<br>TTGAACAGGCGCAGTCTGAGCAGCTT	71	[21]
Borrelia bissettii	rpoB	Forward<br>Reverse<br>Probe	GCAACCAGTCAGCTTTCACAG<br>CAAATCCTGCCCTATCCCTTG<br>AAAGTCCTCCCGGCCCAAGAGCATTAA	87	[21]
Borrelia miyamotoi	glpQ	Forward<br>Reverse<br>Probe	CACGACCCAGAAATTGACACA<br>GTGTGAAGTCAGTGGCGTAAT<br>TCGTCCGTTTTCTCTAGCTCGATTGGG	94	[21]
Borrelia spp.	23S	Forward<br>Reverse<br>Probe	GAGTCTTAAAAGGGCGATTTAGT<br>CTTCAGCCTGGCCATAAATAG<br>AGATGTGGTAGACCCGAAGCCGAGT	73	[21]
Anaplasma marginale	msp1	Forward<br>Reverse<br>Probe	CAGGCTTCAAGCGTACAGTG<br>GATATCTGTGCCTGGCCTTC<br>ATGAAAGCCTGGAGATGTTAGACCGAG	85	[21]
Anaplasma platys	groEL	Forward<br>Reverse<br>Probe	TTCTGCCGATCCTTGAAAACG<br>CTTCTCCTTCTACATCCTCAG<br>TTGCTAGATCCGGCAGGCCTCTGC	75	[21]
Anaphasma phagocytophilum	msp2	Forward<br>Reverse<br>Probe	GCTATGGAAGGCAGTGTTGG<br>GTCTTGAAGCGCTCGTAACC<br>AATCTCAAGCTCAACCCTGGCACCAC	77	[21]
Anaplasma centrale	groEL	Forward<br>Reverse<br>Probe	AGCTGCCCTGCTATACACG<br>GATGTTGATGCCCAATTGCTC<br>CTTGCATCTCTAGACGAGGTAAAGGGG	79	[21]
Anaplasma bovis	groEL	Forward<br>Reverse<br>Probe	GGGAGATAGTACACATCCTTG<br>CTGATAGCTACAGTTAAGCCC<br>AGGTGCTGTTGGATGTACTGCTGGACC	73	[23]
Anaplasma spp.	16S	Forward<br>Reverse<br>Probe	CTTAGGGTTGTAAAACTCTTTCAG<br>CTTTAACTTACCAAACCGCCTAC<br>ATGCCCTTTACGCCCAATAATTCCGAACA	160	[23]
Ehrlichia spp.	16S	Forward<br>Reverse<br>Probe	GCAACGCGAAAAACCTTACCA<br>AGCCATGCAGCACCTGTGT<br>AAGGTCCAGCCAAACTGACTCTTCCG	98	[23]

(Continued)

Targeted TBP	Targeted gene	Identity	Sequence	Amplicon size (bp)	Reference
Ehrlichia canis	gltA	Forward	GACCAAGCAGTTGATAAAGATGG	136	[23]
		Reverse	CACTATAAGACAATCCATGATTAGG		
		Probe	ATTAAAACATCCTAAGATAGCAGTGGCTAAGG		
Neoehrlichia mikurensis	groEL	Forward	AGAGACATCATTCGCATTTTGGA	96	[21]
		Reverse	TTCCGGTGTACCATAAGGCTT		
		Probe	AGATGCTGTTGGATGTACTGCTGGACC		
Rickettsia conorii	23S-5S ITS	Forward	CTCACAAAGTTATCAGGTTAAATAG	118	[21]
		Reverse	CGATACTCAGCAAAATAATTCTCG		
		Probe	CTGGATATCGTGGCAGGGCTACAGTAT		
Rickettsia slovaca	23S-5S ITS	Forward	GTATCTACTCACAAAGTTATCAGG	138	[21]
		Reverse	CTTAACTTTTACTACAATACTCAGC		
		Probe	TAATTTTCGCTGGATATCGTGGCAGGG		
Rickettsia massiliae	23S-5S ITS	Forward	GTTATTGCATCACTAATGTTATACTG	128	[21]
		Reverse	GTTAATGTTGTTGCACGACTCAA		
		Probe	TAGCCCCGCCACGATATCTAGCAAAAA		
Rickettsia helvetica	23S-5S ITS	Forward	AGAACCGTAGCGTACACTTAG	79	[21]
		Reverse	GAAAACCCTACTTCTAGGGGT		
		Probe	TACGTGAGGATTTGAGTACCGGATCGA		
Rickettsia aeschlimannii	ITS	Forward	CTCACAAAGTTATCAGGTTAAATAG	134	[21]
		Reverse	CTTAACTTTTACTACGATACTTAGCA		
		Probe	TAATTTTTGCTGGATATCGTGGCGGGG		
Rickettsia felis	orfB	Forward	ACCCTTTTCGTAACGCTTTGC	163	[23]
		Reverse	TATACTTAATGCTGGGCTAAACC		
		Probe	AGGGAAACCTGGACTCCATATTCAAAAGAG		
Rickettsia spp.	gltA	Forward	GTCGCAAATGTTCACGGTACTT	78	[23]
		Reverse	TCTTCGTGCATTTCTTTCCATTG		
		Probe	TGCAATAGCAAGAACCGTAGGCTGGATG		
Bartonella henselae	pap31	Forward	CCGCTGATCGCATTATGCCT	107	[21]
		Reverse	AGCGATTTCTGCATCATCTGCT		
		Probe	ATGTTGCTGGTGGTGTTTCCTATGCAC		
Bartonella spp.	ssrA	Forward	CGTTATCGGGCTAAATGAGTAG	118	[23]
		Reverse	ACCCCGCTTAAACCTGCGA		
		Probe	TTGCAAATGACAACTATGCGGAAGCACGTC		
Francisella tularensis	tul4	Forward	ACCCACAAGGAAGTGTAAGATTA	76	[21]
		Reverse	GTAATTGGGAAGCTTGTATCATG		
		Probe	AATGGCAGGCTCCAGAAGGTTCTAAGT		
	fopA	Forward	GGCAAATCTAGCAGGTCAAGC	91	[21]
		Reverse	CAACACTTGCTTGAACATTTCTAG		
		Probe	AACAGGTGCTTGGGATGTGGGTGGTG		
Coxiella burnettii	IS1111	Forward	TGGAGGAGCGAACCATTGGT	86	[21]
		Reverse	CATACGGTTTGACGTGCTGC		
		Probe	ATCGGACGTTTATGGGGATGGGTATCC		
	icd	Forward	AGGCCCGTCCGTTATTTTACG	74	[21]
		Reverse	CGGAAAATCACCATATTCACCTT		
		Probe	TTCAGGCGTTTTGACCGGGCTTGGC		
Apycomplexa	18S	Forward	TGAACGAGGAATGCCTAGTATG	104	[23]
		Reverse	CACCGGATCACTCGATCGG		
		Probe	TAGGAGCGACGGGCGGTGTGTAC		
Babesia microti	CCTeta	Forward	ACAATGGATTTTCCCCAGCAAAA	145	[21]
		Reverse	GCGACATTTCGGCAACTTATATA		
		Probe	TACTCTGGTGCAATGAGCGTATGGGTA		

(Continued)

Targeted TBP	Targeted gene	Identity	Sequence	Amplicon size (bp)	Reference
Babesia canis	hsp70	Forward	TCACTGTGCCTGCGTACTTC	87	[21]
		Reverse	TGATACGCATGACGTTGAGAC		
		Probe	AACGACTCCCAGCGCCAGGCCAC		
Babesia ovis	18S	Forward	TCTGTGATGCCCTTAGATGTC	92	[21]
		Reverse	GCTGGTTACCCGCGCCTT		
		Probe	TCGGAGCGGGGTCAACTCGATGCAT		
Babesia bovis	CCTeta	Forward	GCCAAGTAGTGGTAGACTGTA	100	[21]
		Reverse	GCTCCGTCATTGGTTATGGTA		
		Probe	TAAAGACAACACTGGGTCCGCGTGG		
Babesia caballi	rap1	Forward	GTTGTTCGGCTGGGGCATC	94	[21]
		Reverse	CAGGCGACTGACGCTGTGT		
		Probe	TCTGTCCCGATGTCAAGGGGCAGGT		
Babesia venatorum (sp. EU1)	18S	Forward	GCGCGCTACACTGATGCATT	91	[21]
		Reverse	CAAAAATCAATCCCCGTCACG		
		Probe	CATCGAGTTTAATCCTGTCCCGAAAGG		
Babesia divergens	hsp70	Forward	GCGCGCTACACTGATGCATT	91	[21]
		Reverse	CAAAAATCAATCCCCGTCACG		
		Probe	CATCGAGTTTAATCCTGTCCCGAAAGG		
Theileria spp.	18S	Forward	GTCAGTTTTTACGACTCCTTCAG	213	[21]
		Reverse	CCAAAGAATCAAGAAAGAGCTATC		
		Probe	AATCTGTCAATCCTTCCTTTGTCTGGACC		
Hepatozoon spp.	18S	Forward	ATTGGCTTACCGTGGCAGTG	175	[23]
		Reverse	AAAGCATTTTAACTGCCTTGTATTG		
		Probe	ACGGTTAACGGGGGATTAGGGTTCGAT		
Ixodes ricinus	ITS2	Forward	CGAAACTCGATGGAGACCTG	77	[21]
		Reverse	ATCTCCAACGCACCGACGT		
		Probe	TTGTGGAAATCCCGTCGCACGTTGAAC		
Tick spp	16S	Forward	AAATACTCTAGGGATAACAGCGT	99	[23]
		Reverse	TCTTCATCAAACAAGTATCCTAATC		
		Probe	CAACATCGAGGTCGCAAACCATTTTGTCTA		
Dermacentor reticulatus	ITS2	Forward	AACCCTTTTCCGCTCCGTG	83	[21]
		Reverse	TTTTGCTAGAGCTCGACGTAC		
		Probe	TACGAAGGCAAACAACGCAAACTGCGA		
Dermacentor marginatus	ITS2	Forward	GCACGTTGCGTTGTTTGCC	139	[21]
		Reverse	CCGCTCCGCGCAAGAATCT		
		Probe	TTCGGAGTACGTCGAGCTCTAGCAGA		
Escherichia coli	eae	Forward	CATTGATCAGGATTTTTCTGGTGATA	102	[21]
		Reverse	CTCATGCGGAAATAGCCGTTA		
		Probe	ATAGTCTCGCCAGTATTCGCCACCAATACC		
