# Supplementary material for: A three-years assessment of Ixodes ricinus-borne pathogens in a French peri-urban forest
Source: Parasit Vectors. 2019 Nov 21;12:551. doi: 10.1186/s13071-019-3799-7 (PMC6873405; doi:10.1186/s13071-019-3799-7)
Supplement: Supplementary file 2 — Additional file 2: Table S2. Targeted genes and primer sequences used for results confirmation. [file 13071_2019_3799_MOESM2_ESM.pdf]

Additional file 2: Table S2. Targeted genes and primer sequences used for results confirmation.

Targeted genus	Targeted gene	Name	Sequence	Reference
Babesia; Theileria; Hepatozoon	18s rRNA gene	BTH 18S 1st F	GTGAAACTGCGAATGGCTCATTAC	[82]
		BTH 18S 1st R	AAGTGATAAGGTTCACAAAACTTCCC	
		BTH 18S 2nd F	GGCTCATTACAACAGTTATAGTTTATTTG	
		BTH 18S 2nd R	CGGTCCGAATAATTCACCGGAT	
Anaplasma; Ehrlichia	16s rRNA gene	EHR1	GAACGAACGCTGGCGGCAAGC	[83]
		EHR2	AGTA(T/C)CG(A/G)ACCAGATAGCCGC	
		EHR3	TGCATAGGAATCTACCTAGTAG	
		EHR2	AGTA(T/C)CG(A/G)ACCAGATAGCCGC	
Borrelia	flaB	FlaB280F	GCAGTTCARTCAGGTAACGG	[84]
		FlaRL	GCAATCATAGCCATTGCAGATTGT	
		flaB_737F	GCATCAACTGTRGTTGTAACATTAACAGG	
		FlaLL	ACATATTCAGATGCAGACAGAGGT	
Rickettsia	rompB	Rc.rompB.4362p	GTCAGCGTTACTTCTTCGATGC	[85]
		Rc.rompB.4,836n	CCGTACTCCATCTTAGCATCAG	
		Rc.rompB.4,496p	CCAATGGCAGGACTTAGCTACT	
		Rc.rompB.4,762n	AGGCTGGCTGATACACGGAGTAA	
